# Social connectedness in mobile gaming: how family dynamics shape children’s virtual interactions

**DOI:** 10.3389/fpsyt.2026.1850835

**Published:** 2026-06-08

**Authors:** Lu Pang, Siti Ezaleila Mustafa, Jiankun Gong, Yizhi Cheng

**Affiliations:** 1 Department of Media and Communication Studies, Faculty of Arts and Social Sciences, University of Malaya, Kuala Lumpur, Malaysia; 2 School of Preschool Education, Hunan College for Preschool Education, Changde, China

**Keywords:** emotion regulation, family dynamics, mobile gaming, social connectedness, virtual interactions

## Abstract

**Background:**

Mobile gaming is important for children’s social interaction, but its impact on real-life social connectedness depends heavily on family dynamics. How family patterns shape children’s emotional and social experiences around gaming remains underexplored, particularly qualitatively. This study examined how family dynamics influence children’s belonging, emotion regulation, and virtual interactions in mobile gaming.

**Methods:**

Using a phenomenological design, we conducted semi-structured interviews with 20 participants from 10 families across urban, suburban, and rural China. Participants included children (n=10, aged 10-12), parents (n=8, all mothers), and siblings (n=2). All children had played mobile games for ≥6 months. Data were analyzed via thematic analysis (Braun & Clarke). Family patterns were identified based on parental mediation, emotional communication, and children’s sense of connectedness.

**Results:**

Three family patterns emerged. Restrictive-Control Families (4/8) showed high monitoring and low trust, linked to concealment, tension, and social shift to virtual peers. Supportive Co-Play Families (3/8) exhibited shared play, emotional communication, and digital-offline continuity, with children reporting greater resilience and belonging. Sibling-Mediated Families (3 families) featured siblings as companions and emotional buffers, helping manage frustration without direct parental involvement. No severe conflicts or distress were directly caused by gaming in any pattern.

**Conclusions:**

Children’s mobile gaming outcomes are shaped by relational context, not just time. This study identifies three family-level regulatory mechanisms: suppression (restrictive-control), cognitive reappraisal (supportive co-play), and co-regulation (sibling-mediated). These findings extend Social Connectedness Theory, showing how family patterns shape children’s emotion regulation. Supportive co-play and sibling mediation facilitate adaptive regulation and connectedness, while restrictive-control may drive children to virtual spaces. Family-based interventions targeting emotion regulation, not just screen reduction, are recommended. Shifting from “anti-addiction” to “developmental enhancement” offers a safe, practical strategy for integrating gaming into family life.

## Introduction

In the digital era, smartphones and mobile games have become deeply embedded in children’s everyday lives (1). In China, as of June 2025, there were 1.123 billion internet users (79.7% penetration rate), with adolescents aged 10–19 making up 13.7% of all users (2). Moreover, 55.5% of elementary school students use mobile games as a primary platform for social interaction (2024 China Adolescent Internet Usage Survey Report).As mobile gaming has become increasingly widespread, it has evolved beyond a form of entertainment into a social environment in which children interact with peers, build friendships, and develop social skills (3, 4). The 2023 Progress Report on the Protection of Minors in China’s Game Industry further highlights that underage users’ preference for gaming is primarily driven by stress relief and social needs. This indicates that online interaction has become a new form of self-presentation, expression, and need fulfillment for minors (5). However, the 2024 China Adolescent Digital Literacy Report finds that adolescents are relatively weaker in emotion regulation and cultural understanding than in cognitive and behavioral dimensions, highlighting the need for more value−guided digital education.

### The double-edged nature of gaming-related social interaction

Numerous studies have shown that online gaming social interactions have a double-edged sword effect. On the positive side, Most games allow players to exert autonomy over the in-game pursuits, exercise their skill and knowledge while facing challenges, and cultivate social connections (6). Therefore, players can obtain achievement and immersion experiences by playing games, which can enhance players’ positive mood and reduce their negative mood (7–9). More importantly, gaming is often a social activity rather than a solitary one. Players communicate, cooperate, and maintain ties with others through shared play (10, 11). Multiplayer games such as Honor of Kings, for example, may support interactions not only among strangers but also among existing friends, thereby expanding children’s opportunities for peer engagement (12). From this perspective, mobile gaming may function as a meaningful arena for children’s socialization.

However, while virtual social connections may expand quantitatively, overuse can lead to multiple crises. In China, approximately 17.0% of adolescent gamers have been reported to display problematic gaming behaviors (13). Research shows that adolescent boys are more likely to prioritize digital gaming as their primary leisure activity, potentially straining relationships with peers, families, and schools (14). Excessive and problematic gaming can erode adolescents’ real-life interpersonal bonds with family members and peers, impair offline social interaction, and further induce social isolation by diminishing face-to-face communication opportunities (15). Excessive screen time associated with gaming can diminish opportunities for children to engage in meaningful offline social interactions (16). These findings highlight a central tension in the literature: mobile gaming may broaden children’s virtual social worlds while simultaneously weakening aspects of their real-world connectedness.

### Family dynamics as the relational context of gaming

Family context appears to be particularly important in understanding this tension. Research has shown that children raised in more negative parenting environments may become more dependent on electronic devices (17), whereas close parent-child relationships and warm family climates can serve as protective factors against later problematic gaming (18). Parental rule-setting, the timing of gaming initiation, and the emotional tone of family responses to gaming may all shape how children engage with digital play (19). At the same time, most existing work has focused either on gaming behavior itself or on its adverse outcomes, while paying comparatively less attention to the relational processes through which families may buffer risks, reshape children’s experiences, or even enhance the potential benefits of gaming. In other words, we still know relatively little about how family interaction patterns influence whether gaming becomes a source of connection, conflict, or compensatory escape.

### A social connectedness perspective

This study adopts the lens of Social Connectedness Theory (SCT). SCT posits that belongingness is a fundamental human motivation (20). Lee & Robbins, (21) later operationalized social connectedness as a psychological construct reflecting an individual’s sense of interpersonal closeness and perceived relational support. Recent work reaffirms this foundational role while extending its relevance to digital and developmental contexts (22). It is not simply the presence of social ties that matters, but the quality of those ties. High-quality relationships are associated with emotional well-being, social adaptation, and resilience, whereas disrupted or low-quality connections may contribute to loneliness, anxiety, depression, and broader difficulties in adjustment. In childhood and adolescence, social connectedness spans multiple relational domains, including family, peers, school, and community (23). Among these, family connectedness is especially consequential. Strong family bonds have been linked to lower emotional distress, fewer depressive symptoms, and greater resilience in the face of social adversity (24–26). Social Connectedness Theory therefore provides a useful framework for examining how children’s online interactions are shaped by, and in turn may affect, their offline relational worlds.

The framework applies Social Connectedness Theory within the context of children’s development, particularly emphasizing how family dynamics and parent-child relationships influence children’s social outcomes. This theory draws attention to the interactive relationship between family dynamics (e.g., parenting styles, family support) and children’s social connectedness. For instance, family interaction quality significantly influences children’s gaming behaviors and preferences (27). Poor parent-child relationships have been linked to excessive gaming and pathological use (28), and family relational trauma may lead adolescents to seek compensatory social involvement through gaming (29). Conversely, supportive parental mediation may transform gaming from a source of conflict into an opportunity for communication, co-regulation, and relationship building (30). Excessive gaming may also feed back into family life by displacing opportunities for interaction and weakening family functioning (31). Together, these findings suggest a reciprocal process in which gaming both reflects and reshapes family relationships.

### Emotion regulation as an analytical lens

In addition to Social Connectedness Theory, this study positions emotion regulation as a core analytical dimension. Drawing on Gross’s (32) process model, we distinguish five families of emotion regulation strategies: situation selection, situation modification, attentional deployment, cognitive change, and response modulation. In the context of mobile gaming, children may employ these strategies both in real time and over longer periods. Family dynamics shape the availability and effectiveness of these strategies. For example, supportive co-play may scaffold adaptive cognitive reappraisal (“It’s just a game, losing is fine”), whereas harsh restrictive mediation may suppress emotional expression and lead to maladaptive response modulation (e.g., venting frustration through aggressive in-game chat). Thus, emotion regulation serves as the key mediating process that translates family interactions into patterns of virtual social connectedness. We treat emotion regulation not as an outcome but as an analytical lens through which to interpret how family dynamics produce specific in-game social behaviors and emotional experiences, ultimately linking to children’s mental health outcomes.

### The present study

Despite this, family dynamics remain underdeveloped in the literature on children’s gaming and social development. Most studies have emphasized either the negative consequences of gaming or the individual motivations that drive it, while giving less attention to the ways family members interpret, regulate, and participate in children’s digital lives. This omission is consequential because similar gaming behaviors may have very different developmental meanings depending on whether they occur in contexts of trust or conflict, emotional attunement or surveillance, shared participation or relational distance. Moreover, although Social Connectedness Theory has been widely applied to offline social functioning, it has rarely been used to examine how family relationships shape children’s experiences in virtual play environments. There remains a need for research that treats mobile gaming not simply as a technological exposure or risk behavior, but as a socially embedded activity whose consequences depend on family interaction patterns.

A growing body of research has identified three distinctive patterns of family interaction around digital gaming, which inform the typology adopted in this study. First, Restrictive-Control Families are characterized by high levels of parental monitoring, rigid rule-setting, and limited trust around gaming activities. Evidence suggests that such restrictive strategies often lead to reduced parent-child communication, increased family conflict, and children’s concealment behaviors as they seek to reclaim autonomy (33). Moreover, controlling communication styles in rule-setting have been associated with more maladaptive gaming outcomes among adolescents (34). Second, Supportive Co-Play Families reflect a contrasting pattern in which parents engage actively with children’s gaming through co-playing, open discussion, and emotional scaffolding. Parent-child joint play has been found to offer a rich context for emotional bonding and learning, with parents supporting children’s goal achievements even when children possess greater gaming competence (35). A recent intervention study further demonstrated that parental participation in video game-based programs can enhance children’s emotional awareness, regulation, and social competence (36). Co-playing and active mediation have also been linked to enhanced family cohesion and reduced gaming-related tensions. Children find that experiences of shared activities contribute to family cohesion and empathetic engagement. Meanwhile, parental involvement through co-use and active mediation enhances family closeness, security, and solidarity, thereby lowering the potential for gaming-related disputes and relieving gaming-induced emotional tensions in the family (37). Third, Sibling-Mediated Families represent an emerging but less studied pattern in which siblings serve as significant intermediaries in children’s gaming experiences. Siblings growing up together frequently engage in shared digital activities, offering each other solicited or unsolicited guidance, problem-solving support, and collaborative play in the absence of adult presence. Older siblings, in particular, perform multiple mediating roles—playmates, mentors, parental proxies, and emotional shields—uniquely shaping younger children’s media experiences (38). Sibling co-playing has been associated with higher levels of sibling affection (39), suggesting that gaming may provide new opportunities for siblings to connect and co-regulate emotions. Taken together, these three patterns—restrictive-control, supportive co-play, and sibling-mediated—provide a theoretically grounded and empirically supported framework for understanding how family dynamics shape children’s digital gaming experiences.

The present study responds to this need by exploring how family dynamics shape children’s social and emotional experiences in mobile gaming. Using a qualitative design, the study examines how parents, children, and siblings interact around gaming, how emotions are managed in these interactions, and how family processes influence children’s sense of belonging across online and offline contexts. Emotion regulation is a critical yet underexplored mechanism in understanding how family dynamics shape children’s virtual interactions in mobile gaming, directly linking it to adolescent mental health outcomes. Rather than asking whether gaming is uniformly good or bad for children, this study asks how different family interaction patterns condition the meanings and consequences of gaming. Specifically, it addresses three questions:

(1) How do parent-child gaming interactions is associated with children’s sense of belonging?
(2) What role does the family play in moderating the emotional impact of gaming?
(3) How can family dynamics mitigate the negative effects of excessive gaming on children’s social relationships?

By centering family dynamics within children’s gaming experiences, this study aims to extend current discussions of digital play and social development in two ways. First, it shifts the focus from gaming behavior alone to the relational processes through which gaming is interpreted and managed within family life. Second, it uses Social Connectedness Theory to clarify how virtual interaction and offline belonging may be linked through everyday family practices. In doing so, the study seeks to provide a more nuanced account of how mobile gaming may either weaken or strengthen children’s real-world social connectedness, depending on the quality of the family relationships that surround it.

## Method

### Research design

This study adopted a qualitative phenomenological design to explore how family dynamics shape children’s virtual interactions in mobile gaming, with particular attention to social connectedness within the family context. A qualitative approach was appropriate because the study aimed to capture participants’ lived experiences, relational meanings, and interpretations of gaming-related family interactions rather than to test predefined hypotheses. The goal was not statistical generalization, but analytical insight into how family processes shape children’s gaming-related social experiences across varied family contexts.

### Participants and recruitment

The study included 20 participants from 10 families across different regions of China, comprising children, parents, and siblings. Participants were recruited using a combination of purposive and snowball sampling (see **Table 1**). Initially, families were identified through local parent WeChat groups, gaming forums, and school partnerships. Additional families were recruited through participant referrals. Eligibility criteria required that children be between 10 and 13 years old, actively engaged in mobile gaming, and have at least 6 months of experience with popular mobile games, with a minimum of 4–5 hours of gameplay per week. Families were selected not to achieve statistical representativeness, but to capture variation in socioeconomic background, household structure, place of residence, and gaming-related interaction patterns. The final sample included dual-income families, single-parent households, and lower-income families, as well as both urban and non-urban families. Seven families were from urban areas, whereas three were from suburban or rural settings. Children ranged in age from 10 to 13 years, and the participating parents represented diverse occupational backgrounds. Sampling continued until the interviews yielded sufficient depth and recurring relational patterns to support thematic development.

**Table 1:** Demographic characteristics of interview participants.

Participant	Role	Gender	Age	Region	Interview duration(min)
1	Child	Male	12	Suburban	30
2	Child	Female	11	Rural	20
3	Child	Male	12	Suburban	42
4	Child	Male	10	Urban	22
5	Child	Male	12	Urban	33
6	Child	Male	10	Urban	30
7	Child	Male	10	Urban	42
8	Child	Male	12	Urban	25
9	Child	Male	12	Urban	30
10	Child	Male	12	Urban	50
11	Parents	Female	38	Suburban	21
12	Parents	Female	40	Rural	24
13	Parents	Female	37	Suburban	25
14	Parents	Female	39	Urban	30
15	Parents	Female	41	Urban	55
16	Parents	Female	38	Urban	23
17	Parents	Female	40	Urban	45
18	Parents	Female	37	Urban	25
19	Siblings	Male	13	Urban	27
20	Siblings	Female	13	Urban	41

### Data collection procedures

Data were collected between November 2024 and March 2025 through in-depth, semi-structured interviews. Most interviews were conducted online via Tencent Meeting, although some families opted for face-to-face interviews in homes or community centers. Interviews lasted between 20 and 55 minutes and were audio-recorded with participants’ consent. One participant provided written responses because of scheduling constraints. All interviews were transcribed for analysis. Audio recordings were transcribed using iFlytek Hear and subsequently checked for accuracy by a second researcher. To protect confidentiality, all data were anonymized using participant codes (e.g., C01, P01, S01) and stored on encrypted university servers. Participants were informed that participation was voluntary and that they could withdraw from the study at any time without penalty.

### Interview guide

The semi-structured interview guide was developed on the basis of Social Connectedness Theory and refined through three rounds of consultation with experts in child development and communication. The guide covered four broad domains: (1) children’s gaming behaviors, (2) family interactions surrounding gaming, (3) perceived social consequences of gaming, and (4) moderating family processes. Example questions included: “How do you feel when you play games with your family members?”, “How do your parents react when you spend time playing games?”, “Has gaming helped you make new friends outside of school?”, and “How does your family handle disagreements over gaming time?” This structure ensured consistency across interviews while allowing flexibility for participants to elaborate on experiences that were personally salient.

### Data analysis

The data were analyzed using thematic analysis following Braun & Clarke (40). Analysis proceeded in several stages. First, the transcripts were read repeatedly to achieve familiarity with the data. Second, initial open codes were generated to identify recurring meanings, interaction patterns, and emotional responses related to gaming and family life. Third, codes were compared both within families and across families in order to identify convergences, contrasts, and family-specific dynamics. Comparing children’s, parents’, and siblings’ accounts of the same family processes was especially important in refining the analytic categories. Fourth, related codes were clustered into candidate themes, which were then reviewed, refined, and named through iterative discussion among the research team.

The analysis was both inductive and theoretically informed. Themes were grounded in participants’ accounts while also being interpreted in light of Social Connectedness Theory, particularly the ways in which family relationships may support or undermine children’s sense of belonging across online and offline contexts.

### Trustworthiness and reflexivity

Several strategies were used to enhance the trustworthiness of the findings. First, triangulation was employed by comparing perspectives from children, parents, and siblings within the same family. This multi-informant design made it possible to examine both convergent and divergent interpretations of the same gaming-related experiences. Second, member checking was conducted with a subset of participating families, who were invited to review summaries of the preliminary findings and comment on whether the interpretations accurately reflected their experiences. Third, the researchers maintained reflective journals throughout the study to monitor how prior assumptions about children’s digital media use and family relationships might shape data collection and interpretation. Weekly coding meetings were also held to compare coding decisions, discuss ambiguous excerpts, and resolve discrepancies through consensus.

## Results

### Overview of family types

The analysis identified three family patterns that differed in how children’s mobile gaming was regulated, emotionally managed, and incorporated into family life: Restrictive-Control Families, Supportive Co-Play Families, and Sibling-Mediated Families. These patterns were derived from interviews with 20 participants, including 10 children (C01–C10), 8 parents (P01–P08), and 2 siblings (S01–S02). Across these family types, mobile gaming was described not only as a leisure activity but also as a setting in which family authority, trust, emotional support, and belonging were negotiated. The differences across family types were most visible in three areas: how gaming rules were imposed or negotiated, how children’s emotional responses to gaming were handled, and whether gaming strengthened or strained family relationships.

### Restrictive-control families: the impact of high control on emotional and social connections

In restrictive-control families, gaming was primarily managed through strict rules, close monitoring, and unilateral parental authority. In these families, gaming was typically framed as a privilege that had to be tightly controlled rather than as a shared family activity. One parent stated, “Tablet password is mine; 30 minutes only after homework” (P03). Children in these families often described hiding or concealing their gaming behavior. As one child explained, “I hide in the bathroom to play when Mom thinks I’m showering” (C07). A sibling also described the tension surrounding such interactions: “When Dad snatches C07’s phone, they don’t speak for days” (S02).

**Figure 1:**
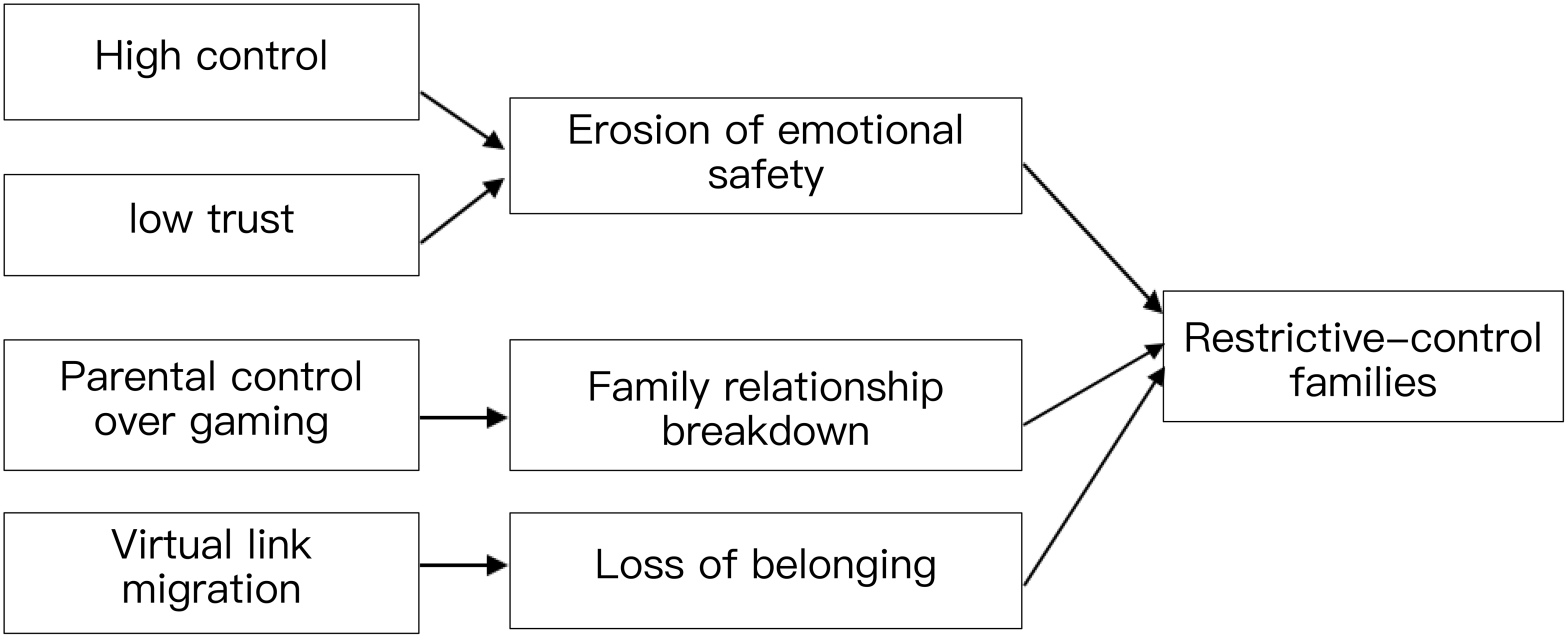
Restrictive-control families: relational strain and eroded belonging.

These accounts indicate that gaming-related control was often accompanied by low levels of trust and strained communication. Rather than discussing gaming openly, family members in this group appeared more likely to engage in conflict, concealment, and withdrawal (see **Figure 1**). Children’s descriptions suggested that gaming became a site of tension within the family, and that emotional distance could widen when gaming rules were enforced through surveillance or punishment. A recurring feature of this family type was the child’s tendency to turn away from the family when gaming-related conflict intensified. Participants described situations in which children sought privacy, avoided direct communication, or became more invested in online interactions after family conflict over gaming. Overall, restrictive-control families were characterized by high parental control, low trust, and weakened emotional openness around gaming.

### Supportive co-play families: strengthening emotional and social bonds through gaming

In supportive co-play families, gaming was incorporated into family interaction in a more collaborative and communicative manner. Parents in these families were involved in gaming not mainly to monitor it, but to understand their children’s interests and share experiences with them. One mother said, “I play Honor of Kings with him—not to be good at it, but to understand what he enjoys” (P05). Children also described these interactions as emotionally supportive. One child noted, “Even if I lose in the game, we talk about it afterward—it helps me calm down” (C04).

**Figure 2:**
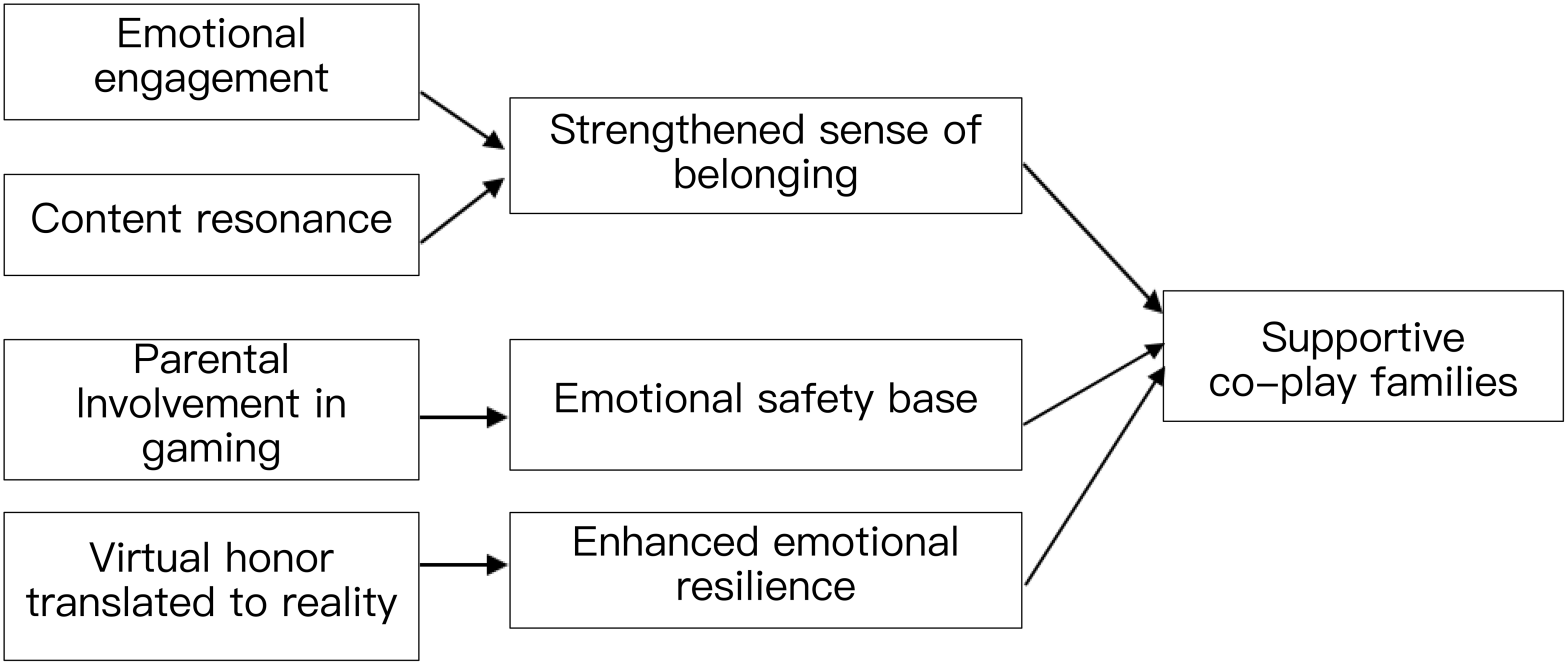
Supportive co-play families: emotional engagement, belonging, and resilience.

In this family type, parents and children often discussed game content, emotional reactions, and gameplay experiences openly. Gaming was treated as a topic of conversation and, in some cases, as a shared activity. Parents did not appear to abandon boundaries, but their involvement was described as engagement-based rather than control-based(see **Figure 2**). Children in these families described feeling understood and emotionally supported, even when they experienced frustration or failure in games. Another visible feature of supportive co-play families was the continuity between online and offline interaction. Gaming experiences were not kept separate from family life; instead, they were brought into everyday communication and emotional exchange. Compared with restrictive-control families, supportive co-play families showed more openness, more emotional dialogue, and greater integration of gaming into family relationships.

### Sibling-mediated families: emotional buffering and peer-like support

In sibling-mediated families, siblings played an important role in shaping children’s gaming experiences and emotional responses. In these families, siblings were described not only as gaming companions but also as sources of reassurance, assistance, and emotional support. One child stated, “When I don’t know how to play, I ask my brother—or we figure out how to win together” (C10). A mother similarly explained, “The older brother often helps the younger one in dungeons; the younger doesn’t cry when he loses because the older one is there” (P07).

**Figure 3:**
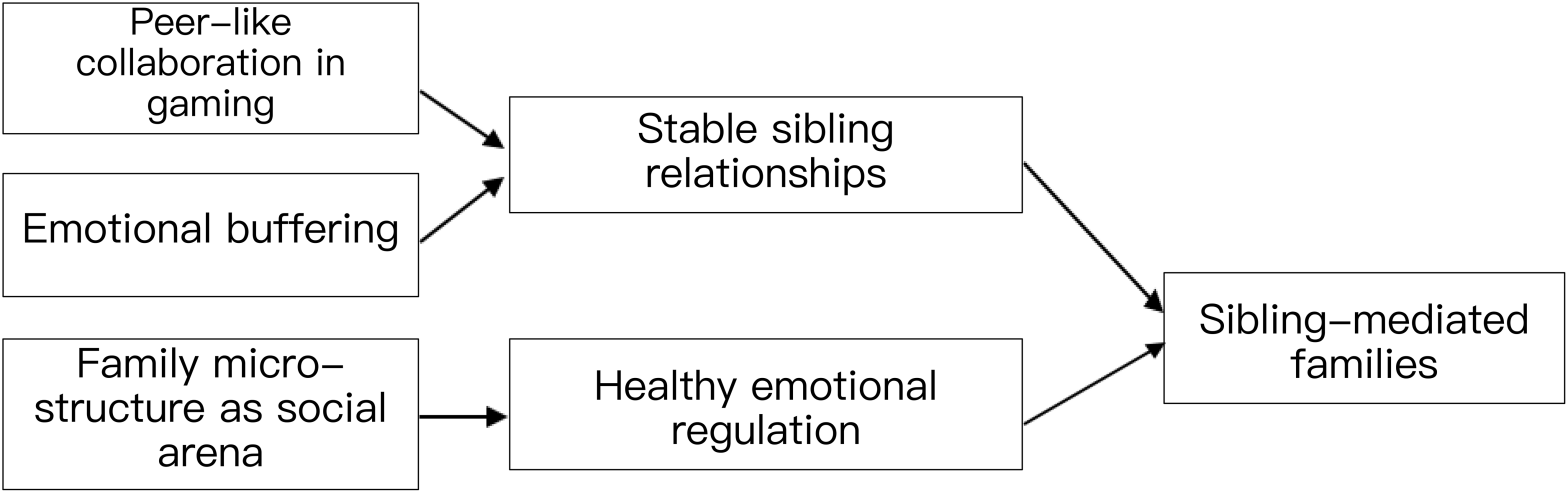
Sibling-mediated families: peer collaboration, emotional buffering, and support.

These accounts suggest that siblings often served as immediate and accessible support figures during gaming. Their role was especially visible when children encountered frustration, confusion, or failure. Instead of facing these experiences alone, younger children often relied on older siblings for guidance and encouragement (see **Figure 3**). Sibling involvement therefore appeared to reduce distress and make gaming experiences more manageable. Compared with parent-led regulation, sibling support was described in a more horizontal and companion-like way. Siblings often helped children solve problems in games, recover from setbacks, and maintain emotional stability during play. In these families, sibling relationships added another layer of support within the household and appeared to help children navigate gaming-related challenges without escalating family conflict.

### Cross-type differences

The three family types differed most clearly in how gaming was positioned within family relationships. In restrictive-control families, gaming was associated with surveillance, secrecy, and tension. In supportive co-play families, gaming functioned as a shared activity that facilitated communication and emotional connection. In sibling-mediated families, siblings provided companionship and emotional buffering during gaming-related challenges. Across these patterns, the activity of gaming itself remained similar, but its relational meaning differed depending on family processes. The findings therefore suggest that children’s gaming experiences were shaped not only by how much they played, but also by how family members responded to, participated in, and interpreted gaming in everyday life.

## Discussion

This study examined how family interaction patterns shape children’s social and emotional experiences in mobile gaming. Across the three family types identified in the analysis—Restrictive-Control Families, Supportive Co-Play Families, and Sibling-Mediated Families—the findings suggest that the social consequences of children’s gaming are shaped less by gaming itself than by the relational context in which gaming is embedded. Mobile gaming emerged not simply as a digital pastime, but as a family-mediated social space in which authority, trust, emotional support, and belonging were negotiated. The findings indicate that family interaction patterns influence whether gaming becomes a source of relational strain, emotional connection, or interpersonal support. This extends existing work on children’s gaming by showing that digital play is not experienced in isolation; rather, its meanings and consequences are filtered through everyday family processes.

### Restrictive control and the redirection of belonging

In restrictive-control families, parents monitor children’s gaming closely, set rules unilaterally, and show little trust. Although these strategies may reduce gaming time in the short term, they often generate unintended relational costs, including secrecy, conflict, and emotional withdrawal. Chang et al. (41) found that such restrictive rules, strict monitoring, and unilateral control are linked to increased problematic gaming and weakened parent–child communication, rather than improved trust or mutual understanding. Consistent with this, children in the present study frequently concealed their gaming, avoided direct conversations, and perceived parental control as surveillance rather than guidance.

Importantly, the present findings indicate that restrictive control may alter not only children’s gaming behavior but also the direction of their search for belonging. When gaming becomes closely associated with punishment, monitoring, and emotional distance, children may become less likely to seek recognition or comfort within the family and more likely to invest in alternative social spaces. This interpretation is consistent with prior work linking problematic gaming to poorer parent-child interaction and relational strain in the family context (27). Rather than reducing children’s need for connection, restrictive control may redirect that need toward online peer environments or gaming-related status systems, especially when family relationships are experienced as low in warmth and accessibility. In this sense, the findings suggest that the issue is not gaming alone, but the relational conditions under which gaming becomes one of the few available spaces for autonomy, competence, and social recognition. From an emotion regulation perspective, restrictive family environments may lead children to rely on maladaptive strategies such as expressive suppression and avoidant coping, which undermine their ability to regulate frustration and distress during gaming, thereby increasing risks for internalizing symptoms and poorer mental health outcomes.

From the perspective of Social Connectedness Theory, restrictive-control families appear to illustrate how weakened emotional accessibility within the family can reshape children’s social investments. Social connectedness is not only a matter of whether children have relationships, but whether those relationships provide a reliable sense of belonging and interpersonal safety. In these families, gaming-related conflict appeared to erode precisely those features of connectedness, thereby making online interaction more attractive as a compensatory relational space. This point strengthens the interpretation that digital engagement may sometimes function less as the cause of relational distance than as a response to it.

### Supportive co-play as emotional communication

By contrast, supportive co-play families demonstrate how gaming can be integrated into family life as a context for emotional communication, shared experience, and relational continuity. In these families, parental involvement was oriented less toward surveillance than toward understanding, participation, and dialogue. This pattern aligns with broader work on active mediation and co-use, which suggests that children benefit not only from boundaries, but also from communicative forms of parental engagement around digital media. For instance, research on Iranian families found that both mothers and fathers tend to mediate children’s digital playtime toward more educational games, using primarily restrictive and active co-playing mediation strategies; notably, fathers may particularly benefit from adopting more technical and active co-playing mediation to further support their children’s digital gameplay at home (42). In the present study, co-play appeared to provide a relatively low-pressure context in which parents could enter children’s digital worlds, discuss frustrations and victories, and respond to emotions in real time. Such interactions seem especially important because they transform gaming from a private or oppositional activity into a shared relational experience.

This pattern can also be understood through Self-Determination Theory. Games tend to be experienced positively when they support the psychological needs for relatedness, autonomy, and competence (43). In supportive co-play families, parental participation may have enhanced all three. Relatedness was supported through shared engagement and emotional dialogue; autonomy was preserved because parents participated without taking over; and competence was acknowledged when children’s gaming experiences were taken seriously rather than dismissed. The present findings therefore suggest that co-play is not merely a behavioral strategy for reducing conflict. It may also function as a relational practice through which children’s online experiences are linked back to offline family belonging. In this sense, co-play appears to help preserve continuity between digital interaction and real-world connectedness rather than allowing the two to diverge. From an emotion regulation lens, co-play functions as emotional buffering: parents model adaptive strategies such as cognitive reappraisal and co-regulate children’s frustration in real time, thereby fostering children’s regulatory skills and promoting resilience against gaming-related emotional distress and subsequent mental health benefits.

More broadly, the supportive co-play pattern suggests that the family can serve as a bridge rather than a barrier to children’s digital lives. When parents approach gaming as an opportunity for communication, children may become more willing to share their digital experiences, including failures, frustrations, and achievements. This matters because social connectedness is not sustained only through protection from risk, but also through repeated experiences of being understood, accompanied, and emotionally received. The present findings therefore extend the literature on family mediation by showing that co-play may foster not only a better family climate, but also a more integrated form of belonging across online and offline settings.

### The distinctive role of siblings

A particularly distinctive contribution of the present study concerns the role of siblings. In sibling-mediated families, siblings were described not only as co-players, but also as emotional buffers (38), informal guides, and readily available support figures during gaming-related challenges. This is theoretically important because much of the literature on children’s digital media use has centered on parents (44), Whereas family research has long recognized siblings as a key subsystem within family dynamics, exerting profound and lasting impacts on children’s emotional regulation, behavioral patterns, and social development (45). The current findings extend that insight into the digital domain by showing that sibling support may shape how children experience gaming, cope with frustration, and remain emotionally regulated during play.

What appears especially important is that sibling support operated differently from parent-led regulation. It was more horizontal, companion-like, and embedded in shared activity. Older siblings often helped younger children interpret setbacks, solve problems, and remain calm in moments of failure. This form of support may be especially effective because it combines emotional reassurance with practical guidance, making it both relationally close and immediately useful. The findings therefore suggest that sibling ties can widen the child’s immediate network of support within the family and may reduce the intensity of gaming-related distress before it escalates into broader family conflict. In this respect, siblings may function as a relational buffer between challenge and dysregulation, helping children stay engaged without becoming overwhelmed. From an emotion regulation perspective, siblings provide a unique context for co-regulation and the rehearsal of adaptive strategies (e.g., situation modification, attentional deployment) in a low-threat, peer-like setting; this informal scaffolding supports children’s emotion regulation development, which in turn protects against gaming-induced emotional dysregulation and contributes to positive mental health outcomes.

This pattern also adds nuance to Social Connectedness Theory. In most applications of the theory, family connectedness is often discussed in relatively global terms. The present findings suggest that connectedness within the family may be internally differentiated: children may experience the family not as a single relational unit, but as a network of distinct ties that operate differently in digital contexts. In households where parent-child regulation is tense or hierarchical, sibling relationships may still provide a more accessible sense of belonging, emotional safety, and co-regulation. This helps explain why sibling-mediated families represent more than a secondary variation on parental mediation; they point to a distinct relational pathway through which children’s online experiences may remain anchored in family support.

### Implications for social connectedness theory

The findings also contribute to Social Connectedness Theory by illustrating how children’s sense of belonging in digital contexts is linked to the quality of family relationships surrounding gaming. Social connectedness is not only a matter of whether children interact with others online, but also of whether those interactions are anchored in supportive offline relationships. Across the three family types, children’s gaming experiences were associated with different forms of belonging: weakened belonging in contexts of high control and distrust, strengthened belonging in contexts of shared engagement, and buffered belonging in contexts of sibling support. Rather than treating virtual and offline connectedness as separate domains, the findings suggest that they are closely intertwined. Family processes appear to shape whether online gaming functions as a compensatory space, a shared relational space, or a supplementary support space. In this respect, the study extends Social Connectedness Theory by showing that the relational meanings of digital interaction depend heavily on the family environment in which that interaction is embedded.

### Practical implications

The findings have several practical implications. At the family level, they suggest that gaming-related conflict may be reduced when parents move away from purely punitive approaches and toward more collaborative forms of mediation. Strategies such as jointly negotiated gaming rules, open discussion about children’s emotional experiences in games, and appropriate forms of parental engagement may help preserve trust while maintaining boundaries. The findings also point to the potential value of involving siblings in family-based approaches to children’s digital media use, particularly in households where sibling relationships are already supportive. More broadly, the results suggest that interventions targeting children’s gaming should focus not only on screen time or rule enforcement, but also on the relational climate in which gaming occurs.

### Limitations and future directions

Several limitations should be noted. First, the study was based on a relatively small qualitative sample and was not intended to provide statistical generalization. Although the sample captured variation in family background and interaction patterns, most participating families were urban, and some family forms—such as intergenerational caregiving households or more diverse single-parent arrangements—were underrepresented. Future research could examine whether similar family patterns emerge across a wider range of household structures and cultural settings. Second, the study did not differentiate systematically among game genres. Competitive, cooperative, and creative games may invite different forms of family involvement and emotional response. Future studies could explore whether the family processes identified here vary depending on the type of game being played. Third, because the study relied on retrospective interview data, future research could benefit from more observational or real-time methods. For example, digital ethnography, screen-recorded family play sessions, or diary-based designs may provide richer insight into how gaming-related family interactions unfold moment to moment. Fourth, the study employed a combination of purposive and snowball sampling. While snowball sampling helped reach families with diverse gaming experiences, this method may introduce selection bias, as initial participants tend to refer others with similar backgrounds or views, potentially limiting the diversity of emergent themes. The reliance on snowball recruitment also means that families with very different or less visible gaming practices may have been missed. Acknowledging this limitation, future qualitative research could use more targeted stratified sampling or community-wide mapping to capture a broader spectrum of family dynamics.

## Conclusion

This study shows that children’s mobile gaming experiences are shaped not only by the games they play, but also by the family relationships surrounding those experiences. Restrictive-control families were marked by tension, concealment, and diminished trust; supportive co-play families by emotional communication and relational continuity; and sibling-mediated families by peer-like support and emotional buffering. These patterns suggest that mobile gaming is best understood as a socially embedded activity whose consequences depend on family interaction processes. By placing family dynamics at the center of children’s digital play, the study offers a more relational account of how gaming may either weaken or strengthen children’s real-world social connectedness.

## Data Availability

The original contributions presented in the study are included in the article/supplementary material. Further inquiries can be directed to the corresponding author.
